# Mitochondrial DNA Copy Number, but Not Haplogroup, Confers a Genetic Susceptibility to Leprosy in Han Chinese from Southwest China

**DOI:** 10.1371/journal.pone.0038848

**Published:** 2012-06-18

**Authors:** Dong Wang, Ling-Yan Su, A-Mei Zhang, Yu-Ye Li, Xiao-An Li, Ling-Ling Chen, Heng Long, Yong-Gang Yao

**Affiliations:** 1 Key Laboratory of Animal Models and Human Disease Mechanisms of the Chinese Academy of Sciences & Yunnan Province, Kunming Institute of Zoology, Kunming, Yunnan, China; 2 The First Affiliated Hospital of Kunming Medical College, Kunming, Yunnan, China; 3 Yuxi City Center for Disease Control and Prevention, Yuxi, Yunnan, China; 4 Wenshan Institute of Dermatology, Wenshan, Yunnan, China; 5 Graduate School of the Chinese Academy of Sciences, Beijing, China; Colorado State University, United States of America

## Abstract

**Background:**

Leprosy is a chronic infectious disease caused by *Mycobacterium leprae*, an unculturable pathogen with an exceptionally eroded genome. The high level of inactivation of gene function in *M. leprae*, including many genes in its metabolic pathways, has led to a dependence on host energy production and nutritional products. We hypothesized that host cellular powerhouse - the mitochondria - may affect host susceptibility to *M. leprae* and the onset of clinical leprosy, and this may be reflected by mitochondrial DNA (mtDNA) background and mtDNA copy number.

**Methods:**

We analyzed the mtDNA sequence variation of 534 leprosy patients and 850 matched controls from Yunnan Province and classified each subject by haplogroup. mtDNA copy number, taken to be proportional to mtDNA content, was measured in a subset of these subjects (296 patients and 231 controls) and 12 leprosy patients upon diagnosis.

**Results:**

Comparison of matrilineal components of the case and control populations revealed no significant difference. However, measurement of mtDNA copy number showed that lepromatous leprosy patients had a significantly higher mtDNA content than controls (*P* = 0.008). Past medical treatments had no effect on the alteration of mtDNA copy number.

**Conclusions:**

Our results suggested that mtDNA content, but not haplogroup, affects leprosy and this influence is limited to the clinical subtype of lepromatous leprosy.

## Introduction

Leprosy is a chronic infectious disease caused by an obligate intracellular bacillus, *Mycobacterium leprae*. According to the WHO, the total of newly registered leprosy patients in 2009 reached 211,903 and many regions in the world have not achieved the leprosy elimination goal [1]. In the regions that had achieved the elimination of leprosy, there are many new features and challenges concerning case finding [2]. Evidently, leprosy remains and will continue to be, a public health problem [1].

The genome of *M. leprae* has been well analyzed during the past decade [3]–[4]. A comparison with *M. tuberculosis*, has shown that more than half of the functional genes are inactivated or have became pseudogenes in the *M. leprae* genome [3]–[5]. These inactivated genes, many of which are predicted to be actively involved in metabolic pathways, have resulted in *M. leprae* being an intracellular parasite. The bacterium has a long half-life and has yet to be cultured *in vitro* [4], [6]. During the long evolutionary erosion of its genome, *M. leprae* has developed a dependence on host energy production and nutritional products and as a result the parasitic life and adaptation might have shaped host genetic susceptibility to leprosy [5], [7], [8].

Mitochondria play a key role in cellular energy supply, *in vivo* generation of reactive oxygen species (ROS), regulation of apoptosis and aging, and participation in innate immunity [9], [10]. The mitochondrion contains its own DNA, mitochondrial DNA (mtDNA), which encodes 13 respiratory chain subunits, 2 ribosomal RNAs, and 22 tRNAs. Due to the specific characteristics of mtDNA, e.g. maternal inheritance, absence of recombination and high mutation rate, each mtDNA lineage accumulated mutations during evolution and a group of mtDNAs that share certain mtDNA ancestral variants can be shown to belong to distinguishable haplogroups [11], [12]. Previous studies have shown that the mtDNA haplogroup background affected genetic susceptibility to many diseases, including maternally inherited diseases [e.g. Leber’s hereditary optic neuropathy, LHON [13], [14]], metabolic diseases [e.g. diabetes [15], [16]], infectious diseases [e.g. acquired immune deficiency syndrome (AIDS) [17] and sepsis [18]]. Whether the mtDNA haplogroup background confers a genetic susceptibility to leprosy has not been studied.

Each cell has hundreds to thousands of copies of mtDNA molecules, and the alteration of mtDNA content has long been recognized to be affected by the genetic background and factors in the physiological environment, such as thyroid hormone level [19]. mtDNA content was said to be involved in tumorigenesis (e.g. renal cell carcinoma [20] and non-Hodgkin lymphoma [21]) that had a shift of energy production from oxidative phosphorylation to glycolysis. Accumulating evidence showed that mtDNA copy number control is very important for mitochondrial biogenesis and normal cellular function [22]. Moreover, loss of mtDNA copy number was also reported as the cause of mtDNA depletion syndrome [23].

In this study, we hypothesized that erosion of the *M. leprae* genome during its evolution has led to a strong dependence on host energy production and nutritional products, and would confer a genetic susceptibility to leprosy. By dissecting the matrilineal genetic structure of case and control populations and analyzing the mtDNA content in mitochondria, which act as host cellular powerhouse and are actively involved in critical pathways for innate immunity [9], [10], we may have a method of testing this hypothesis. Our comparison of mtDNA haplogroup distribution frequency and mtDNA copy number in leprosy patients and healthy controls showed that mtDNA copy number, but not haplogroup, was associated with lepromatous leprosy.

## Materials and Methods

### Ethics Statement

Written informed consents conforming to the tenets of the Declaration of Helsinki were obtained from each participant prior to the study. The institutional review board of the Kunming Institute of Zoology approved this study.

### Subjects

The study included 534 treated leprosy patients (390 males and 144 females, age from 17 to 98 years) with detailed clinical data and 850 matched normal controls (467 males and 383 females, age from 3 to 91 years) without any history of leprosy, HIV infection and tuberculosis from nine regions of Yuxi Prefecture, Yunnan Province in Southwest China (Figure S1). The Yuxi Prefecture of Yunnan Province (north latitude 23° 19′–24° 53′, longitude 101° 16′–103° 9′) is historically an endemic region of leprosy [2] and contains eight counties (Jiangchuan, Chengjiang, Tonghai, Huaning, E’shan, Yimen, Xinping, and Yuanjiang) and one district (Hongta). In our recent study, we have reported the leprosy diagnosis, control, and treatment results in this region from 1952 to 2008 [2].

Patients were diagnosed on the basis of clinical presentation and histopathological features, as well as bacteriological index (if available), following the approach described in Li et al. [2]. The clinical data for those patients were described in our recent study [2], and the onset age of the patients ranged from 2 to 67 years. 283 of the patients had been diagnosed with multibacillary leprosy (MB, including lepromatous [LL], borderline-lepromatous [BL] and mid-borderline leprosy [BB] based on the Ridley–Jopling classification [24]) and 249 with paucibacillary leprosy (PB, including tuberculoid [TT] and borderline-tuberculoid [BT] leprosy on the basis of the Ridley–Jopling classification) according to the latest WHO case definition [25]. Two samples had an unclear classification of leprosy subtype due to lack of detailed information.

392 patients had been treated by Dapsone (DDS) monotherapy and/or Rifampicin (RFP), of which 34 patients experienced relapse. 142 patients had been treated with the WHO multi-drug therapy (MDT). All the relapsed patients were subsequently treated by MDT. Disability levels were graded according to the revised WHO grading system [26]. Samples from 12 newly diagnosed patients prior to any treatment were collected from Wenshan, Yunnan (7 males and 5 females, age from 13 to 80 year) (Figure S1).

### Sequencing mtDNA Control Region and Haplogroup Classification

Genomic DNA was extracted from peripheral blood by using the AxyPrep™ Blood Genomic DNA Miniprep Kit (Axygen, USA). The mtDNA control region sequence of each patient and control was amplified by using primer pair (L15594, 5′-CGCCTACACAATTCTCCGATC-3′/H901, 5′-ACTTGGGTTAATCGTGTGACC-3′ or H650, 5′-GAAAGGCTAGGACCAAACCTA-3′) [27]. PCR products were purified and sequenced following the same method described in our recent study [27], [28]. Sequence variation was scored relative to the revised Cambridge reference sequence (rCRS) [29]. Each mtDNA was classified into the smallest named haplogroup according to the available mtDNA phylogeny for East Asian [11], [30] by employing the same strategy in our previous studies [13], [28]. For samples which could not be unambiguously classified based on the mtDNA control region sequence variation, PCR- restriction fragment length polymorphism (RFLP) analysis for haplogroup-specific coding region variant(s) was performed to justify the assignment [13], [28].

### Measurement of mtDNA Copy Number

Relative mtDNA copy number was measured by the fluorescence-based quantitative real-time PCR as previously described in our recent study [31]. In brief, primer pair L394, 5′-CACCAGCCTAACCAGATTTC-3′/H475, 5′-GGGTTGTATTGATGAGATTAGT-3′ was used for measuring the mtDNA and the amplification of the single-copy nuclear β-globin gene (HBB502f, 5′-CTATGGGACGCTTGATGT-3′/HBB614r, 5′-GCAATCATTCGTCTGTTT-3′) was used for normalization. The ratio of mtDNA to nuclear DNA, the relative mtDNA copy number, reflects the relative mtDNA content per cell. The amplification assays were performed with SYBR® Premix Ex Taq™ II kit (TaKaRa Biotechnology Co. Ltd. [Dalian, China]) according the manufacturer’s manual on the MyiQ2 system (BioRad Laboratories, Hercules, CA, USA).

### Statistical Analysis

Pearson’s chi-square test with a one degree of freedom was used to assess the significance of differences observed for haplogroup distribution frequencies between leprosy patients and controls. The Fisher’s exact test (two tailed) was applied to those cases with cell counts below five. Principal component analysis (PCA) was performed based on the mtDNA haplogroup distribution frequency to show the overall clustering pattern of leprosy and control populations from Yuxi with reported Han Chinese populations across China (Zhang et al. [28] and references therein) by using POPSTR software (http://harpending.humanevo.utah.edu/popstr/).

The mtDNA copy number was compared between the case group and the control group by using Student’s *t* test. Difference of mtDNA copy number among three or more groups was evaluated by one-way analysis of variance (ANOVA). Dunnett’s test was further used for multiple comparisons between any of the two groups. SPSS 15.0 was used for all statistical analysis. A *P* value <0.05 was considered statistically significant.

## Results

### Lack of Association of mtDNA Haplogroup with Leprosy

The patient and control populations from nine regions of the Yuxi Prefecture had various sample size, ranging from 16 to 164 individuals (Figure S1). We therefore pooled all the cases and controls from different regions of Yuxi as one case population and one control population for analysis, respectively, and excluded the 12 samples from Wenshan. All 1384 subjects (534 patients and 850 controls) could be classified into known haplogroups in East and South Asians [11], [30] based on mtDNA variation, with a few lineages with unassigned M*, N* and R* status (Table S1). There was no significant difference in mtDNA haplogroup frequency between the patients and controls (Table 1). When the leprosy patients were classified into two groups (MB group [N = 288) and PB group [N = 256]) according to their clinical features, no haplogroup was found to be associated with leprosy subtype relative to controls (Table 1). To further demonstrate the similarity of matrilineal genetic structures of leprosy patient and control populations from Yuxi, we constructed a PC map for these two populations, together with reported Han Chinese populations across China (Zhang et al. [28] and references therein). The leprosy patient and control populations were clustered together, suggesting no essential difference between them (Figure 1). This result further indicated the absence of any association of mtDNA haplogroup with leprosy.

**Table 1:** Haplogroup frequencies and Pearson’s chi-square test in leprosy patients and controls from Yuxi, Yunnan Province in Southwest China.

Haplogroup#	Controls	Leprosy patients				MB patients				PB patients			
	No.	No.	*P*-value*	OR	95% CI	No.	*P*-value*	OR	95% CI	No.	*P*-value*	OR	95% CI
C	45	19	0.134	1.515	0.876–2.620	8	0.089	1.922	0.895–4.127	10	0.416	1.336	0.663–2.691
Z	24	20	0.341	0.747	0.408–1.365	10	0.544	0.793	0.375–1.680	10	0.339	0.694	0.327–1.473
CZ	72	39	0.436	1.175	0.783–1.762	18	0.256	1.362	0.798–2.326	20	0.839	1.055	0.629–1.769
M7b	39	18	0.267	1.379	0.780–2.436	12	0.807	1.086	0.560–2.104	6	0.127	1.948	0.815–4.655
M7c	8	8	0.345	0.625	0.233–1.675	4	0.508	0.663	0.198–2.218	4	0.485	0.582	0.174–1.949
M7	47	27	0.703	1.099	0.676–1.787	17	0.763	0.916	0.517–1.622	10	0.350	1.393	0.693–2.799
M8a	17	6	0.214	1.796	0.704–4.584	2	0.185	2.867	0.658–12.488	4	1.000	1.250	0.417–3.750
M9a	24	12	0.512	1.264	0.627–2.549	5	0.330	1.615	0.611–4.275	7	0.992	1.004	0.428–2.360
M11	5	4	0.740	0.784	0.210–2.933	1	1.000	1.669	0.194–14.344	3	0.391	0.485	0.115–2.045
M12	6	7	0.256	0.535	0.179–1.601	2	1.000	0.999	0.200–4.977	5	0.069	0.347	0.105–1.146
M25	5	4	0.740	0.784	0.210–2.933	0	–	–	–	4	0.124	0.362	0.097–1.360
M71	9	4	0.776	1.418	0.434–4.628	2	0.741	1.504	0.323–7.001	2	1.000	1.322	0.284–6.157
M74	5	4	0.740	0.784	0.210–2.933	2	1.000	0.831	0.160–4.309	2	0.660	0.731	0.141–3.790
D4	100	58	0.607	1.094	0.776–1.542	37	0.558	0.886	0.592–1.327	21	0.140	1.448	0.884–2.371
D5	28	16	0.758	1.103	0.591–2.058	8	0.698	1.171	0.527–2.599	7	0.703	1.178	0.508–2.729
D	128	75	0.604	1.085	0.797–1.476	45	0.733	0.938	0.648–1.357	29	0.183	1.339	0.870–2.059
G	53	38	0.520	0.868	0.564–1.336	21	0.485	0.830	0.491–1.402	17	0.737	0.908	0.516–1.598
A	80	44	0.457	1.157	0.787–1.700	23	0.515	1.174	0.723–1.907	21	0.638	1.128	0.682–1.865
N9a	23	13	0.757	1.115	0.560–2.220	8	0.914	0.956	0.423–2.162	5	0.539	1.357	0.511–3.608
B4	100	77	0.150	0.791	0.575–1.089	44	0.098	0.724	0.494–1.063	33	0.527	0.873	0.573–1.330
B5	43	25	0.752	1.085	0.655–1.798	10	0.293	1.455	0.721–2.934	15	0.549	0.831	0.454–1.523
B	148	108	0.604	1.085	0.797–1.476	57	0.302	0.836	0.595–1.175	51	0.257	0.814	0.571–1.162
F1	123	82	0.652	0.933	0.689–1.263	48	0.311	0.828	0.575–1.193	34	0.746	1.070	0.711–1.611
F2	14	14	0.210	0.622	0.294–1.315	6	0.601	0.773	0.294–2.031	8	0.121	0.504	0.209–1.217
F3	8	4	0.776	1.259	0.377–4.201	4	0.508	0.663	0.198–2.218	0	–	–	–
F	154	111	0.219	0.843	0.642–1.107	63	0.125	0.773	0.555–1.075	48	0.658	0.922	0.643–1.322
R9b	14	4	0.222	2.219	0.727–6.777	0	–	–	–	4	1.000	1.026	0.335–3.145
R9	172	117	0.456	0.904	0.694–1.178	64	0.393	0.868	0.627–1.201	53	0.718	0.938	0.663–1.327
Others	56	32	0.658	1.106	0.707–1.733	16	0.577	1.177	0.664–2.087	16	0.927	1.027	0.578–1.824

* *P*-value was calculated by Pearson’s chi-square test; Fisher's exact test was used when haplogroup occurred in less than five individuals.

# Haplogroup occurred in less than four individuals in the entire case or control population and those unassigned M*, N* and R* mtDNAs were pooled together. We presented these nested haplogroups according to their phylogenetic positions, e.g. D contains D4, D5, D6 and D*.

**Figure 1:**
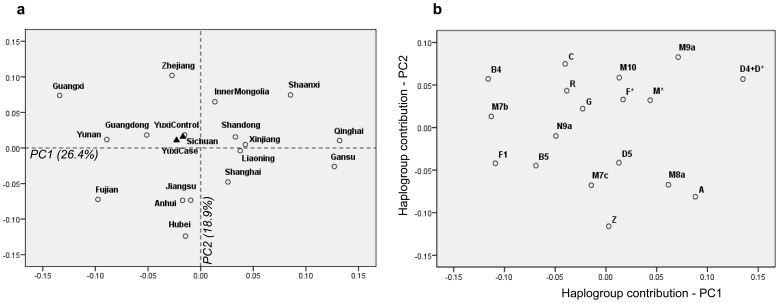
Principal component analysis of leprosy patient and control populations from Yuxi and reported Han Chinese populations across China. (a) PC map of Han regional populations based on mtDNA haplogroup frequencies (>1%). The patient (YuxiCase) and control (YuxiControl) populations from Yuxi were marked by triangles, whereas the reported Han Chinese populations (Zhang et al. [28] and references therein) were marked by open circles. (b) Plot of the haplogroup contribution to the first and second PCs.

### Increase of mtDNA Copy Number in Lepromatous Leprosy Patients

To test whether alterations of mtDNA copy number affected leprosy, we measured the mtDNA copy number in samples from 296 randomly selected patients (224 males and 72 females, age from 35 to 98 year) and 231 matched controls (142 males and 89 females, age from 13 to 85 year) from Yuxi. Twelve newly diagnosed patients prior to treatment from Wenshan were also analyzed to determine any potential effect of drug treatment on mtDNA content (Table 2). Twenty-two samples were measured twice to assess the reproducibility of our technique and we observed similar results in the two replicates.

**Table 2:** Demographic information and mtDNA content in the case and control groups.

Variable	Yuxi cases (N = 296)^a^		Wenshan cases (N = 12)		Controls (N = 231)	
	No. (%)	mtDNA content (mean ± SD)	No. (%)	mtDNA content (mean ± SD)	No. (%)	mtDNA content (mean ± SD)
**Sex**						
Male	224 (75.7)	64.18±38.30	7 (58.3)	44.41±12.38	142 (61.5)	58.93±19.69
Female	72 (24.3)	64.94±36.91	5 (41.7)	55.28±25.52	89 (38.5)	60.31±18.72
*P*-value†		0.882		0.416		0.597
**Age, year**						
< mean age*	139 (47.0)	58.73±33.57	7 (58.3)	42.90±9.14	122 (52.8)	59.14±19.87
> mean age	157 (53.0)	69.36±40.83	5 (41.7)	57.39±26.24	109 (47.2)	59.83±18.71
*P*-value†		0.015		0.293		0.786
**WHO classification** #						
MB	163 (55.4)	66.97±37.88	5 (41.7)	41.29±12.89	–	–
PB	131 (44.6)	61.10±37.98	7 (58.3)	54.41±21.22	–	–
*P*-value†		0.187		0.250		–
**Treatment**						
MDT	102 (34.6)	65.51±40.53	–	–	–	–
DDS/RFP	193 (65.4)	63.73±36.64	–	–	–	–
*P*-value†		0.703		–		–

* The mean age varied in each group of subjects (Mean ± SD, Yuxi case: 61.1±12.3; Wenshan case: 34.5±21.3; Control: 39.9±16.8).

† Student's unpaired t test (two-sided) was used to quantify the differences.

# Two cases from Yuxi had unclear leprosy types were excluded. All leprosy patients from Wenshan received no treatment prior to blood sample collection.

**Figure 2:**
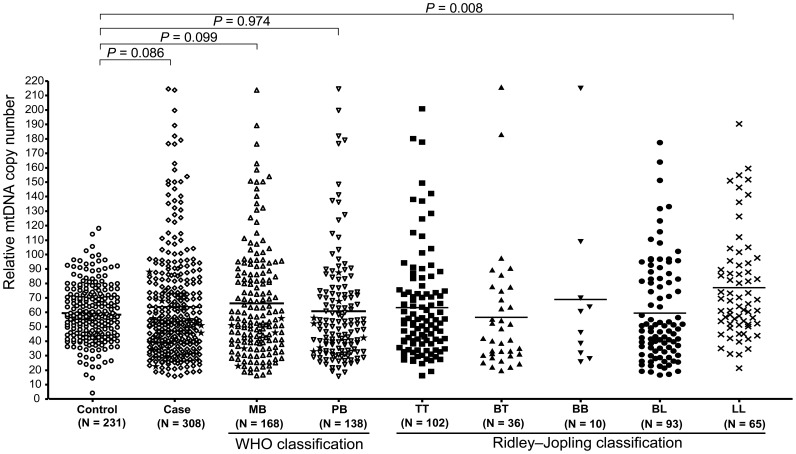
Comparison of the relative mtDNA copy number in leprosy patients (n = 308) and matched healthy controls (n = 231). Student’s unpaired *t* test was used to quantify the statistical difference between the leprosy patient and control groups. Leprosy patients were either grouped into multibacillary leprosy (MB) and paucibacillary leprosy (PB) according to the latest WHO case definition [25] or into lepromatous (LL), borderline-lepromatous (BL), mid-borderline leprosy (BB), borderline-tuberculoid (BT), and tuberculoid (TT) based on the Ridley–Jopling classification [24]. One-way analysis of variance (ANOVA) of the mean values was used to analyze the difference of mtDNA copy number among groups (for comparison among MB, PB and control groups, *P* = 0.089; for comparison among LL, BL, BB, BT, TT and control groups, *P* = 0.003); the Dunnett’s test was further used for multiple comparisons between any of the two groups. The 12 untreated leprosy samples from Wenshan were marked with stars in MB, PB and case groups, and the two patients with unclear classification were excluded from the analysis when we grouped the patients into MB and PB groups.

As shown in Figure 2, there was a slight increase of mtDNA copy number in all leprosy patients (Mean ± SD, 63.76±37.45) compared to control subjects (59.46±19.29) (*P* = 0.086). When we grouped the leprosy patients according to their case definition (MB versus PB), we found that the observed higher level of mtDNA copy number in leprosy patients was mainly contributed by MB patients (versus control, *P* = 0.099), although there was no significant difference of mtDNA copy number between MB group (66.21±37.62; n = 168) and PB group (60.90±37.40; n = 138) (*P* = 0.219; the 2 cases from Yuxi with an unclear leprosy type were excluded). Further assignment of patients according to the Ridley–Jopling classification system [24] showed that patients with lepromatous leprosy had a significantly higher level of mtDNA copy number compared to the control sample (*P* = 0.008), whereas the other types of leprosy showed no significant difference with the controls (Figure 2). We observed a significantly elevated level of mtDNA copy number in the cured patients from Yuxi with advanced age (>61 year) compared to patients with age younger than 61 (*P* = 0.015; Table 2). However, there was no essential change of mtDNA copy number with age in the controls and this observation was consistent with previous reports [20], [32]. The increased level of mtDNA copy number in Yuxi cases with advanced age was not simply caused by a greater number of LL cases in this group (32% of LL cases had an age <61 years and 68% of LL patients had an age >61 years), as we still observed a higher level of mtDNA copy number in cured patients with advanced age than in patients with young age after we excluded all LL patients (*P* = 0.049). Because there was no statistical difference in age between the MB and PB groups and among the five patient groups according to the Ridley–Jopling classification system, the increased mtDNA content in LL patients was unlikely to be caused by age difference. A comparison of mtDNA copy number in samples with different haplogroup status showed that haplogroup background did not affect mtDNA content (Table S2). Taken together, the above observed difference should be attributed to the lepromatous leprosy subtype.

To test whether long-term treatment to cure leprosy had an effect on mtDNA copy number, we measured the level of mtDNA copy number in patients who had received the different drug treatments of DDS and/or RFP (63.73±36.64) and MDT (65.51±40.53), and found that both groups showed similar values. Moreover, the level of mtDNA copy number in the 12 leprosy patients prior to any medical treatment was within the range of that observed in the cured patients and control subjects from Yuxi, suggesting no effect of past treatment on alteration of mtDNA copy number (Figure 2).

**Figure 3:**
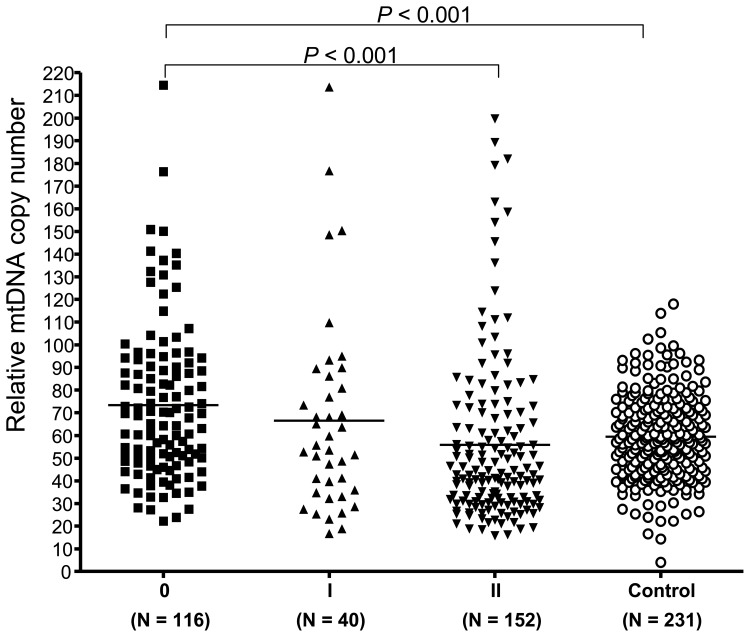
Relative levels of mtDNA copy number in leprosy patients with different grades of disability. One-way analysis of variance (ANOVA) of the mean values was used to analyze the difference of mtDNA copy number among groups (with a *P* value <0.001) and the Dunnett’s test was used for multiple comparisons between any of the two groups.

We followed the WHO disability grading [26], which scored different level of severity of impairment on limbs (hands and feet) and eyes, to group all patients into three groups. Intriguingly, we found that patients with no anesthesia, no visible deformity and damage, and no visual loss and other eye problem (Grade 0) had a higher level of mtDNA copy number (73.33±33.98) relative to the control (*P*<0.001). There was a decreasing tendency for the relative level of mtDNA copy number in all patients with increasing disability grading (Grade I, 66.53±43.48; Grade II, 55.73±36.69) (Figure 3). However, this tendency was not correlated with disability grade observed in LL cases (Grade 0, 83.32±32.1; Grade I, 64.0±15.7; and Grade II, 72.4±42.7).

## Discussion

In recent years, many human disorders have been reported to be associated with mtDNA haplogroup [13]–[18], [28] and/or alteration of mtDNA content [20], [21], [33]. Due to the intrinsic characteristics of the eroded genome of *M. leprae*, we would expect that host mtDNA background and content would confer genetic susceptibility to leprosy. Moreover, accumulating evidence has suggested that mitochondria play an essential role in the innate immunity response to microbial infection [9], [10], [34], and this innate immune response may play a crucial role in determining whether mycobacterial infection progresses to the clinical disease of leprosy [35], [36]. Analyses of mtDNA haplogroup distributions in case-control studies have reported positive associations of mtDNA variants/haplogroups with infectious complications and sepsis [17], [18], [37]–[39], although there are negative reports, e.g. lack of association with mtDNA haplogroups and meningococcal disease [40].

In this study, we analyzed mtDNA haplogroup distribution and mtDNA copy number variation in a large cohort of leprosy patients and matched controls from Southwest China, to test whether mtDNA variation confers a genetic susceptibility to leprosy. We found that mtDNA content, but not mtDNA haplogroup, was associated with lepromatous leprosy. To our knowledge, this is the first report to link mtDNA variation with genetic susceptibility to leprosy.

A previous study reported that sepsis was associated with reduction of mtDNA content in circulating monocytes and lymphocytes, which influence the innate immune response to infections [33], and longitudinal increases of mtDNA copy number in blood cells was associated with survival in critically ill patients [41]. The LL type of leprosy is known to harbor a greater number of bacilli and be predisposed to low cell mediated immunity [42]. Whether the higher mtDNA copy number in LL patients negatively impacts on the immune response to *M. leprae* infection remains elusive. We speculated that the higher level of mtDNA content in LL patients might be associated with the development of lepromatous leprosy, which was accompanied by the secretion of cytokines and possibly increased oxidative stress. Indeed, a previous study by Lee et al. [43] have shown that cellular mtDNA content and mitochondrial mass would increase in response to oxidative stress. However, we must confess that this speculation could not explain the fact that we failed to observe a graded difference in mtDNA copy number from polar tuberculoid to lepromatous leprosy, as expected from the increasing bacterial load. Because there was no statistical difference in the ages of the LL group and the other patient groups, the elevated level of mtDNA copy number in LL patients could not be simply explained by the potential age difference. Moreover, we still observed a higher level of mtDNA copy number in patients with advanced age after we excluded all LL cases. The disability in leprosy patients was directly related to host immunological reactions and early access to treatment [42]. We observed a decreasing tendency for the relative level of mtDNA copy number in patients with increasing disability grading. The exact reason for this difference awaits further study.

Some antibiotics could cause decreased cell growth, reduced mitochondrial mass, impaired respiratory complex activities and mitochondrial protein synthesis, but these inhibition effects could be reversed after stopping the antibiotic [44]. The majority of our patients who received treatment were cured more than a decade ago. We found that these patients once treated with DDS and/or RFP had similar level of mtDNA content compared with those who received MDT, suggesting that mtDNA content might not be affected by different drugs. Moreover, the mtDNA content in leprosy patients prior to any medical treatment was within the range that was observed in cured patients and control subjects (Figure 2), albeit the overall level of mtDNA content was lower in these untreated patients (Table 2) and the sample size was very small. All these observations seemed to suggest that past medical treatments might not have an effect on mtDNA content. Because we did not collect patients who are undergoing therapy, we do not know whether there is a change of mtDNA content upon drug treatment. In addition, it would be worthwhile to compare mtDNA copy number at the time of relapse and the time of last treatment in the same patients, to test whether there are alterations of mtDNA copy number.

In summary, we found no mtDNA haplogroup that was associated with leprosy but observed an increased mtDNA copy number in patients who had developed lepromatous leprosy. This observation could be compatible with the notion of a higher cellular energy supply facilitating the survival and persistence of *M. leprae in vivo*. On this point, our current result is compatible with our hypothesis that host cellular powerhouse - the mitochondria - may affect the susceptibility to *M. leprae* and the development of clinical leprosy. Functional characterization of mitochondria in patients with leprosy should be performed to independently validate our findings.

## Supporting Information

Figure S1 **Map showing sampling location and regional sample size.**
(PDF)

Table S1 **mtDNA sequence variation and haplogroup classification of 534 leprosy patients and 850 normal controls from Yuxi, Yunnan, Southwest China.**
(XLS)

Table S2 **mtDNA copy number in leprosy patients (n = 296) and controls (n = 231) with different haplogroup status.**
(PDF)

## References

[1] WHO (2010). Global leprosy situation, 2010.. Wkly Epidemiol Rec.

[2] Li Y-Y, Li X-A, He L, Wang D, Chen W-Y (2011). Trends in new leprosy case detection over 57 years (1952–2008) in Yuxi, Yunnan Province of Southwest China.. Lepr Rev.

[3] Monot M, Honoré N, Garnier T, Zidane N, Sherafi D (2009). Comparative genomic and phylogeographic analysis of *Mycobacterium leprae*.. Nat Genet.

[4] Cole ST, Eiglmeier K, Parkhill J, James KD, Thomson NR (2001). Massive gene decay in the leprosy bacillus.. Nature.

[5] Misch EA, Berrington WR, Vary JC, Hawn TR (2010). Leprosy and the human genome.. Microbiol Mol Biol Rev.

[6] Britton WJ, Lockwood DN (2004). Leprosy.. Lancet.

[7] Alcaïs A, Mira M, Casanova JL, Schurr E, Abel L (2005). Genetic dissection of immunity in leprosy.. Curr Opin Immunol.

[8] Alter A, Grant A, Abel L, Alcaïs A, Schurr E (2011). Leprosy as a genetic disease.. Mamm Genome.

[9] McWhirter SM, TenOever BR, Maniatis T (2005). Connecting mitochondria and innate immunity.. Cell.

[10] Arnoult D, Soares F, Tattoli I, Girardin SE (2011). Mitochondria in innate immunity.. EMBO Rep.

[11] Kong Q-P, Bandelt H-J, Sun C, Yao Y-G, Salas A (2006). Updating the East Asian mtDNA phylogeny: a prerequisite for the identification of pathogenic mutations.. Hum Mol Genet.

[12] Torroni A, Achilli A, Macaulay V, Richards M, Bandelt H-J (2006). Harvesting the fruit of the human mtDNA tree.. Trends Genet.

[13] Ji Y, Zhang A-M, Jia X, Zhang Y-P, Xiao X (2008). Mitochondrial DNA haplogroups M7b1'2 and M8a affect clinical expression of Leber hereditary optic neuropathy in Chinese families with the m.11778G>A mutation.. Am J Hum Genet.

[14] Hudson G, Carelli V, Spruijt L, Gerards M, Mowbray C (2007). Clinical expression of Leber hereditary optic neuropathy is affected by the mitochondrial DNA-haplogroup background.. Am J Hum Genet.

[15] Achilli A, Olivieri A, Pala M, Hooshiar Kashani B, Carossa V (2011). Mitochondrial DNA backgrounds might modulate diabetes complications rather than T2DM as a whole.. PLoS One.

[16] Fuku N, Park KS, Yamada Y, Nishigaki Y, Cho YM (2007). Mitochondrial haplogroup N9a confers resistance against type 2 diabetes in Asians.. Am J Hum Genet.

[17] Hendrickson SL, Hutcheson HB, Ruiz-Pesini E, Poole JC, Lautenberger J (2008). Mitochondrial DNA haplogroups influence AIDS progression.. AIDS.

[18] Baudouin SV, Saunders D, Tiangyou W, Elson JL, Poynter J (2005). Mitochondrial DNA and survival after sepsis: a prospective study.. Lancet.

[19] Moraes CT (2001). What regulates mitochondrial DNA copy number in animal cells?. Trends Genet.

[20] Xing J, Chen M, Wood CG, Lin J, Spitz MR (2008). Mitochondrial DNA content: its genetic heritability and association with renal cell carcinoma.. J Natl Cancer Inst.

[21] Lan Q, Lim U, Liu C-S, Weinstein SJ, Chanock S (2008). A prospective study of mitochondrial DNA copy number and risk of non-Hodgkin lymphoma.. Blood.

[22] Clay Montier LL, Deng JJ, Bai Y (2009). Number matters: control of mammalian mitochondrial DNA copy number.. J Genet Genomics.

[23] Rötig A, Poulton J (2009). Genetic causes of mitochondrial DNA depletion in humans.. Biochim Biophys Acta.

[24] Ridley DS, Jopling WH (1966). Classification of leprosy according to immunity. A five-group system.. Int J Lepr Other Mycobact Dis.

[25] WHO (1982). WHO Technical Report Series, no. 675.. Geneva: World Health Organization; Chemotherapy of leprosy for control programmes.

[26] WHO (1988). WHO expert committee on leprosy. Sixth report.. Geneva.

[27] Yao Y-G, Childs RW, Kajigaya S, McCoy JP, Young NS (2007). Mitochondrial DNA sequence heterogeneity of single CD34+ cells after nonmyeloablative allogeneic stem cell transplantation.. Stem Cells.

[28] Zhang A-M, Jia X, Bi R, Salas A, Li S (2011). Mitochondrial DNA haplogroup background affects LHON, but not suspected LHON, in Chinese patients.. PLoS One.

[29] Andrews RM, Kubacka I, Chinnery PF, Lightowlers RN, Turnbull DM (1999). Reanalysis and revision of the Cambridge reference sequence for human mitochondrial DNA.. Nat Genet.

[30] Kong Q-P, Sun C, Wang H-W, Zhao M, Wang W-Z (2011). Large-scale mtDNA screening reveals a surprising matrilineal complexity in east Asia and its implications to the peopling of the region.. Mol Biol Evol.

[31] Bi R, Zhang A-M, Zhang W, Kong Q-P, Wu B-L (2010). The acquisition of an inheritable 50-bp deletion in the human mtDNA control region does not affect the mtDNA copy number in peripheral blood cells.. Hum Mutat.

[32] Miller FJ, Rosenfeldt FL, Zhang C, Linnane AW, Nagley P (2003). Precise determination of mitochondrial DNA copy number in human skeletal and cardiac muscle by a PCR-based assay: lack of change of copy number with age.. Nucleic Acids Res.

[33] Pyle A, Burn DJ, Gordon C, Swan C, Chinnery PF (2010). Fall in circulating mononuclear cell mitochondrial DNA content in human sepsis.. Intensive Care Med.

[34] Wang C, Liu X, Wei B (2011). Mitochondrion: an emerging platform critical for host antiviral signaling.. Expert Opin Ther Targets.

[35] Modlin RL (2010). The innate immune response in leprosy.. Current Opinion in Immunology.

[36] Montoya D, Modlin RL (2010). Learning from leprosy: insight into the human innate immune response.. Adv Immunol.

[37] Huebinger RM, Gomez R, McGee D, Chang LY, Bender JE (2010). Association of mitochondrial allele 4216C with increased risk for sepsis-related organ dysfunction and shock after burn injury.. Shock.

[38] Yang Y, Shou Z, Zhang P, He Q, Xiao H (2008). Mitochondrial DNA haplogroup R predicts survival advantage in severe sepsis in the Han population.. Genet Med.

[39] García-Álvarez M, Guzmán-Fulgencio M, Berenguer J, Micheloud D, Campos Y (2011). European mitochondrial DNA haplogroups and liver fibrosis in HIV and hepatitis C virus coinfected patients.. AIDS.

[40] Salas A, Fachal L, Marcos-Alonso S, Vega A, Martinon-Torres F (2009). Investigating the role of mitochondrial haplogroups in genetic predisposition to meningococcal disease.. PLoS One.

[41] Côté HC, Day AG, Heyland DK (2007). Longitudinal increases in mitochondrial DNA levels in blood cells are associated with survival in critically ill patients.. Crit Care.

[42] Walker SL, Lockwood DN (2006). The clinical and immunological features of leprosy.. Br Med Bull.

[43] Lee H-C, Yin P-H, Lu C-Y, Chi C-W, Wei Y-H (2000). Increase of mitochondria and mitochondrial DNA in response to oxidative stress in human cells.. Biochem J.

[44] Soriano A, Miró O, Mensa J (2005). Mitochondrial toxicity associated with linezolid.. N Engl J Med.

