# Supplementary material for: Mitochondrial DNA Copy Number, but Not Haplogroup, Confers a Genetic Susceptibility to Leprosy in Han Chinese from Southwest China
Source: PLoS One. 2012 Jun 18;7(6):e38848. doi: 10.1371/journal.pone.0038848 (PMC3377694; doi:10.1371/journal.pone.0038848)
Supplement: Figure S1 — Map showing sampling location and regional sample size. (PDF) [file pone.0038848.s001.pdf]

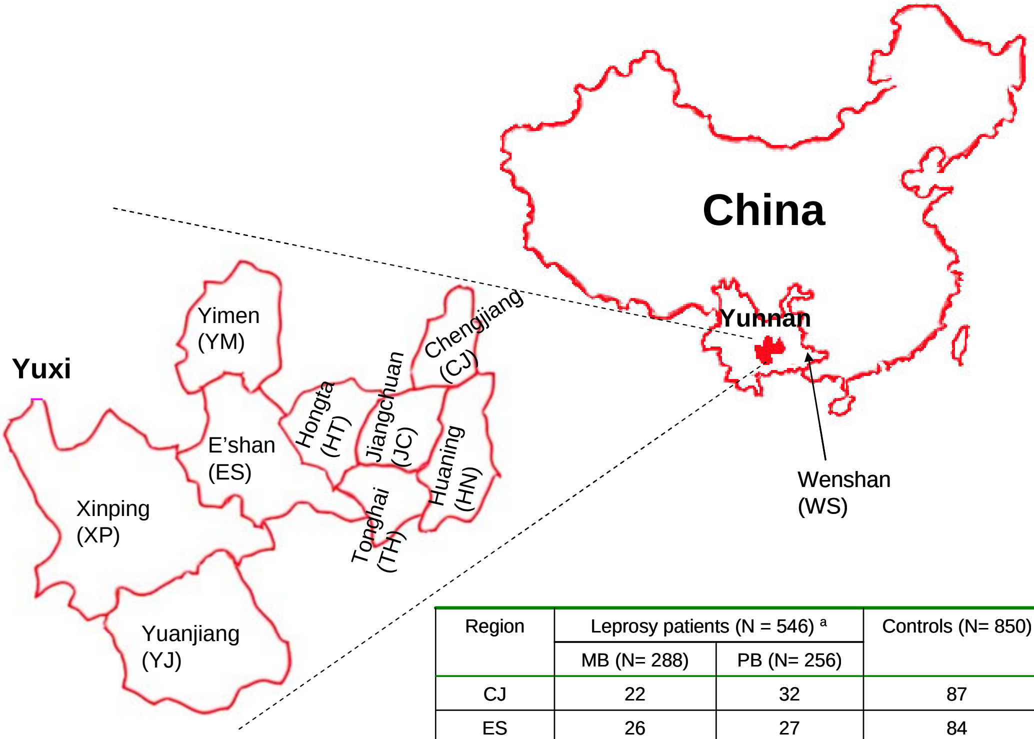

Region	Leprosy patients (N = 546) [a]		Controls (N= 850)
	MB (N= 288)	PB (N= 256)	
CJ	22	32	87
ES	26	27	84
HN	71	27	164
HT	36	31	107
JC	36	40	123
TH	39	41	123
XP	13	5	26
YJ	11	4	22
YM	29	42	114
WS	5	7	0

[a] Two leprosy patients (one in HN and one in YJ) had unclear leprosy type and were excluded.
