# Supplementary material for: Mitochondrial DNA Copy Number, but Not Haplogroup, Confers a Genetic Susceptibility to Leprosy in Han Chinese from Southwest China
Source: PLoS One. 2012 Jun 18;7(6):e38848. doi: 10.1371/journal.pone.0038848 (PMC3377694; doi:10.1371/journal.pone.0038848)
Supplement: Table S2 — mtDNA copy number in leprosy patients (n = 296) and controls (n = 231) with different haplogroup status. (PDF) [file pone.0038848.s003.pdf]

Table S2. mtDNA copy number in leprosy patients (n=296) and controls (n=231) with different haplogroup status

Haplogroup*	Leprosy patients		Controls		*P*-value[#]
	No.	Mean ± SD	No.	Mean ± SD	
C	11	56.14 ± 35.45	11	51.72 ± 12.32	0.700
Z	9	64.61 ± 49.30	8	57.09 ± 11.60	0.668
M7b	8	72.18 ± 40.66	12	56.86 ± 19.45	0.271
M9a	7	50.25 ± 21.26	5	47.63 ± 16.95	0.824
D4	29	75.41 ± 49.58	30	59.44 ± 22.59	0.115
D5	10	48.34 ± 14.83	10	55.60 ± 12.98	0.259
G	20	58.02 ± 30.05	16	54.99 ± 24.96	0.734
A	25	56.08 ± 28.73	20	65.96 ± 18.16	0.188
N9a	9	70.60 ± 52.77	10	66.98 ± 20.06	0.841
B4	48	70.09 ± 34.27	26	69.51 ± 17.13	0.936
B5	18	57.78 ± 28.74	8	58.96 ± 15.77	0.915
F	58	72.71 ± 43.57	40	61.00 ± 18.04	0.071
P-value[‡]		0.379		0.132	

* Haplogroups occurred in less than four individuals were excluded.

[#] *P*-value was calculated by student's unpaired *t* test (two tailed).

[‡] *P*-value of one-way analysis of variance (ANOVA) for the difference of mtDNA copy number among different haplogroups.
